# Evaluation and activity of new porphyrin-peptide cage-type conjugates for the photoinactivation of *Mycobacterium abscessus*

**DOI:** 10.1128/spectrum.00006-24

**Published:** 2024-04-15

**Authors:** Matthéo Alcaraz, Sébastien Lyonnais, Chandramouli Ghosh, John Jairo Aguilera-Correa, Sébastien Richeter, Sébastien Ulrich, Laurent Kremer

**Affiliations:** 1 Centre National de la Recherche Scientifique UMR 9004, Institut de Recherche en Infectiologie de Montpellier (IRIM), Université de Montpellier, Montpellier, France; 2 CEMIPAI, UAR3725, CNRS, Université de Montpellier, Montpellier, France; 3 Institut des Biomolécules Max Mousseron (IBMM), Université of Montpellier, CNRS, ENSCM, Montpellier, France; 4 Institut Charles Gerhardt Montpellier (ICGM), Université de Montpellier, CNRS, ENSCM, Montpellier, France; 5 INSERM, IRIM, Montpellier, France; Johns Hopkins University School of Medicine, Baltimore, Maryland, USA

**Keywords:** *Mycobacterium abscessus*, photodynamic inactivation, photosensitizer, therapeutic activity, atomic force microscopy

## Abstract

*Mycobacterium abscessus* is increasingly recognized as an emerging opportunistic pathogen causing severe lung diseases and cutaneous infections. However, treatment of *M. abscessus* infections remains particularly challenging, largely due to intrinsic resistance to a wide panel of antimicrobial agents. New therapeutic alternatives are urgently needed. Herein, we show that, upon limited irradiation with a blue-light source, newly developed porphyrin-peptide cage-type photosensitizers exert a strong bactericidal activity against smooth and rough variants of *M. abscessus* in planktonic cultures and in biofilms, at low concentrations. Atomic force microscopy unraveled important morphological alterations that include a wrinkled and irregular bacterial surface. The potential of these compounds for a photo-therapeutic use to treat *M. abscessus* skin infections requires further evaluations.

**IMPORTANCE:**

*Mycobacterium abscessus* causes persistent infections and is extremely difficult to eradicate. Despite intensive chemotherapy, treatment success rates remain very low. Thus, given the unsatisfactory performances of the current regimens, more effective therapeutic alternatives are needed. In this study, we evaluated the activity of newly described porphyrin-peptide cage-type conjugates in the context of photodynamic therapy. We show that upon light irradiation, these compounds were highly bactericidal against *M. abscessus in vitro*, thus qualifying these compounds for future studies dedicated to photo-therapeutic applications against *M. abscessus* skin infections.

## OBSERVATION

*Mycobacterium abscessus* is a rapidly growing mycobacterial species, often isolated in patients with cystic fibrosis (CF) or with other lung defects (1). In the context of lung infections, *M. abscessus* has emerged as an important opportunistic pathogen associated with significant mortality (2). It causes also cutaneous infections (3), which remains very difficult to treat, essentially due to its intrinsic resistance to many antibiotics, and requires prolonged periods of treatment, often associated with side effects. Thus, there is an unmet clinical need for new therapeutic approaches with better efficacy, while shortening the duration of the treatments.

**Fig 1:**
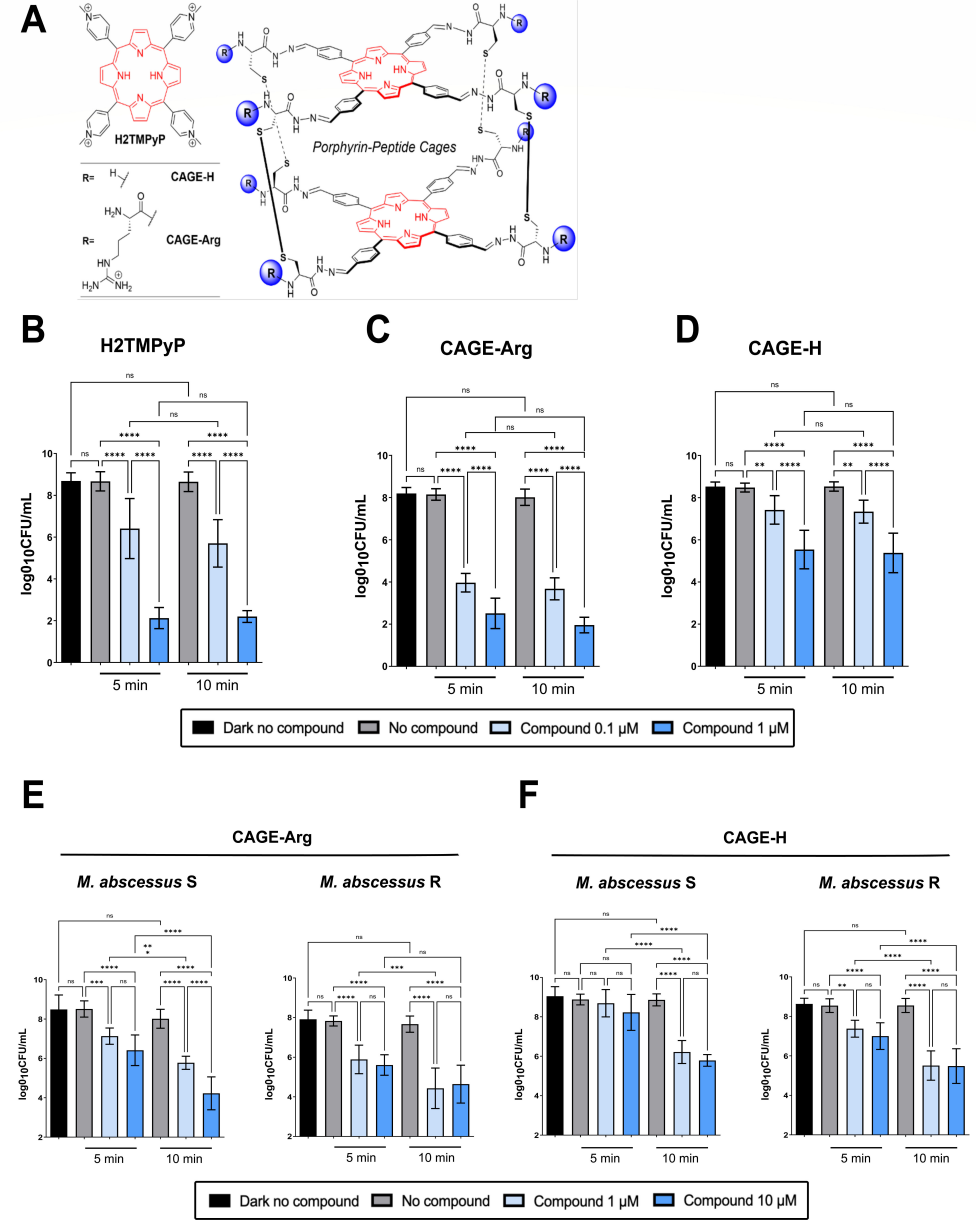
Structures of the porphyrin-based PS used in this study and activity against *Escherichia coli* (B–D) and *M. abscessus* (E and F). (A) Structures of H2TMPyP, CAGE-H, and CAGE-Arg. (B–D) Cultures of *E. coli* were exposed 0.1 (light blue bars) and 1 µM (dark blue bars) of H2TMPyP (B), CAGE-Arg (C), or CAGE-H (D) for 30 min prior to irradiation with a blue light source for 5 or 10 min. Colony-forming unit (CFU) quantification was determined immediately after light irradiation. Untreated (black bars) and irradiated bacteria without PS (gray bars) were included as controls. Data are of three independent experiments in triplicate and were analyzed using one-way analysis of variance (ANOVA) test or with Kruskal-Wallis test. **P ≤ 0.01; ****P ≤ 0.0001; ns, non-significant. (E and F) Cultures of *M. abscessus* S and R were exposed to 1 or 10 µM of CAGE-Arg (E) or CAGE-H (F) for 30 min prior to irradiation with a blue light source for 5 or 10 min. CFU quantification was determined immediately after light irradiation. Untreated (black colums) and irradiated bacteria without PS (gray colums) were included as controls. The error bars represent the standard deviation. Data are of three independent experiments in triplicate and were analyzed using one-way ANOVA test. **P ≤ 0.01; ***P ≤ 0.001; ****P ≤ 0.0001; ns, non-significant.

In this context, photodynamic therapy (PDT), initially tested for the treatment of tumor cells, has appeared as a possible alternative to control infections. PDT relies on (organic) photosensitizers (PS) (4), which, following light irradiation, produces reactive oxygen species (ROS), leading the rapid photo-oxidation of various biomolecules, such as lipids, proteins, or nucleic acids, leading to rapid cell death. Porphyrins have been used as PS for PDT with antibiotic activity against several human pathogens (5). In particular, cationic porphyrins promote strong electrostatic interactions with the polyanionic DNA backbone and membrane proteins (6, 7). The tetra-cationic *N*-methylated porphyrin (H2TMPyP) (Fig. 1A) has been reported to kill *M. abscessus* subsp. *massiliense* and *Mycobacterium fortuitum* (8), and there is current interest in exploring complex supramolecular and dynamic covalent assemblies as new antibiotics (9, 10). Herein, we explored porphyrin-peptide cage-type conjugates, precisely the positively charged CAGE-Arg and neutral CAGE-H (Fig. 1A) (11), as new architectures to inactivate *M. abscessus* in planktonic cultures and biofilms.

Preliminary experiments showed that long periods of exposure to the blue light was associated with a decrease in the viability of *Escherichia coli* (not shown), prompting us to identify experimental conditions for short periods of irradiation to limit the toxic effect. *E. coli* Stellar cultures at a 600-nm optical density (OD_600_) of 0.8 were washed twice with a 0.9% NaCl, dispensed in 96-well plates, and incubated either with the PS control (H2TMPyP) (12), CAGE-Arg, or CAGE-H at 0.1 µM or 1 µM for 30 min at 37°C in the dark. Then, plates were irradiated for 5 or 10 min using a led blue light source (EvoluChem Led Light HCK1012-01-12, 425 nm, 33 mW/cm^2^) kept at a 10 cm distance from the wells. Bacteria were diluted in a 10-fold bank dilution of phosphate buffered saline (PBS) and plated onto lysogeny broth (LB) agar to quantify CFU after overnight incubation at 37°C. Under these conditions, incubation with 0.1 µM H2TMPyP, followed by irradiation with a blue light for 5 min, was associated with a 2-Log reduction in the CFUs as compared to bacteria kept in the dark. This effect was further exacerbated by an additional 4-Log decrease in the CFUs with 1 µM H2TMPyP (Fig. 1B; Table S1). Similar results were observed when increasing the duration of the treatment to 10 min (Fig. 1B). This underscores the PDT activity of H2TMPyP against *E. coli,* validating our experimental setup. We next assessed and observed a dose-dependent activity of CAGE-Arg (Fig. 1C; Table S1) and CAGE-H (Fig. 1D; Table S1), although the neutral cage exerted a reduced activity as compared to the cationic cage. As for H2TMPyP, increasing the duration of photoactivation from 5 to 10 min did not improve the PDT activity of the compounds. In the absence of irradiation, the compounds did not affect bacterial viability (not shown). We evaluated the effect of CAGE-Arg and H2TMPyP, the most active compound against the planktonic state, on *E. coli* biofilm (Supplemental Materials and Methods, Fig. S1; Table S2). CAGE-Arg at 1 µM for 10 min provoked a 2-Log reduction in the CFUs (Fig. S1A), while H2TMPyP at 1 µM was associated with a 4-Log decrease in the CFUs at both time points (Fig. S1B).

We next tested the activity of CAGE-Arg and CAGE-H against the smooth (S) and the rough (R) variants of *M. abscessus* CIP104536^T^ (13), characterized by the presence or absence of cell surface glycopeptidolipids, respectively, and differing in their virulence profile; the R strain exhibiting a more pathogenic behavior in mice (14), zebrafish (15), and CF patients (16). Cultures were grown in Middlebrook 7H9 supplemented with 10% OADC enrichment and tyloxapol 0.025% (7H9^OADC/Ty^) to reach an OD_600nm_ = 1, washed twice with 0.9% NaCl to prevent scavengers of ROS in 7H9^OADC/Ty^, and washed with a 0.9% NaCl solution containing 0.025% tyloxapol. Bacterial suspensions were sonicated for 3 min in a bath sonicator to remove clumps and dispensed in 96-well plates. PS were added at a concentration of 1 or 10 µM for 30 min in the dark at 37°C prior to irradiation for 5 or 10 min with the blue led light. Serial dilutions in PBS supplemented with 0.025% of tyloxapol were prepared, sonicated for 3 min, and plated onto LB agar, and CFU were counted after 3 days at 37°C. A robust PDT activity of CAGE-Arg (at 1 and 10 µM) was found against *M. abscessus* S and R (Fig. 1E; Table S3), while in general, neutral CAGE-H exhibited a weaker activity than cationic CAGE-Arg (Fig. 1F; Table S3) at comparable times of irradiation and concentrations. Overall, this indicates that CAGE-Arg and CAGE-H exert potent PDT activity upon photoactivation at 425 nm and that a 5-min irradiation is sufficient to kill *M. abscessus* in planktonic state.

*M. abscessus* can also be associated with biomaterial-related infections, where the main pathological mechanism involves biofilm formation, contributing also to persistence, rendering eradication even more challenging (17). We, thus, assessed the activity of H2TMPyP on *M. abscessus* S biofilms growing in a leukocyte lysate medium, which promotes mycobacterial biofilm formation (18) and then focused on CAGE-Arg, since it displays a better killing activity than CAGE-H *in vitro*. H2TMPyP reduced 79.5% (0.688 Log_10_, *P* = 0.0164) of mycobacterial viability at 10 µM after 5 min of irradiation inside the biofilm and this reduction was 79.7% (0.6932 Log_10_, *P* = 0.0151) and 86.8% (0.8784 Log_10_, *P* = 0.0005) at 1 and 10 µM, respectively, upon irradiation for 10 min (Fig. S2B; Table S4). Irradiation of CAGE-Arg reduced bacterial viability by 74.7% (0.5962 Log_10_, *P* = 0.0115) and 89.1% (0.961 Log_10_, *P* < 0.0001) at 1 and 10 µM after 5 min, while this reduction was 83.2% (0.7743 Log_10_, *P* = 0.0003) and 95.4% (1.334 Log_10_, *P* < 0.0001) at 1 and 10 µM, respectively, after 10 min of irradiation. This qualifies CAGE-Arg for a photo-therapeutic use to treat *M. abscessus* skin infections.

**Fig 2:**
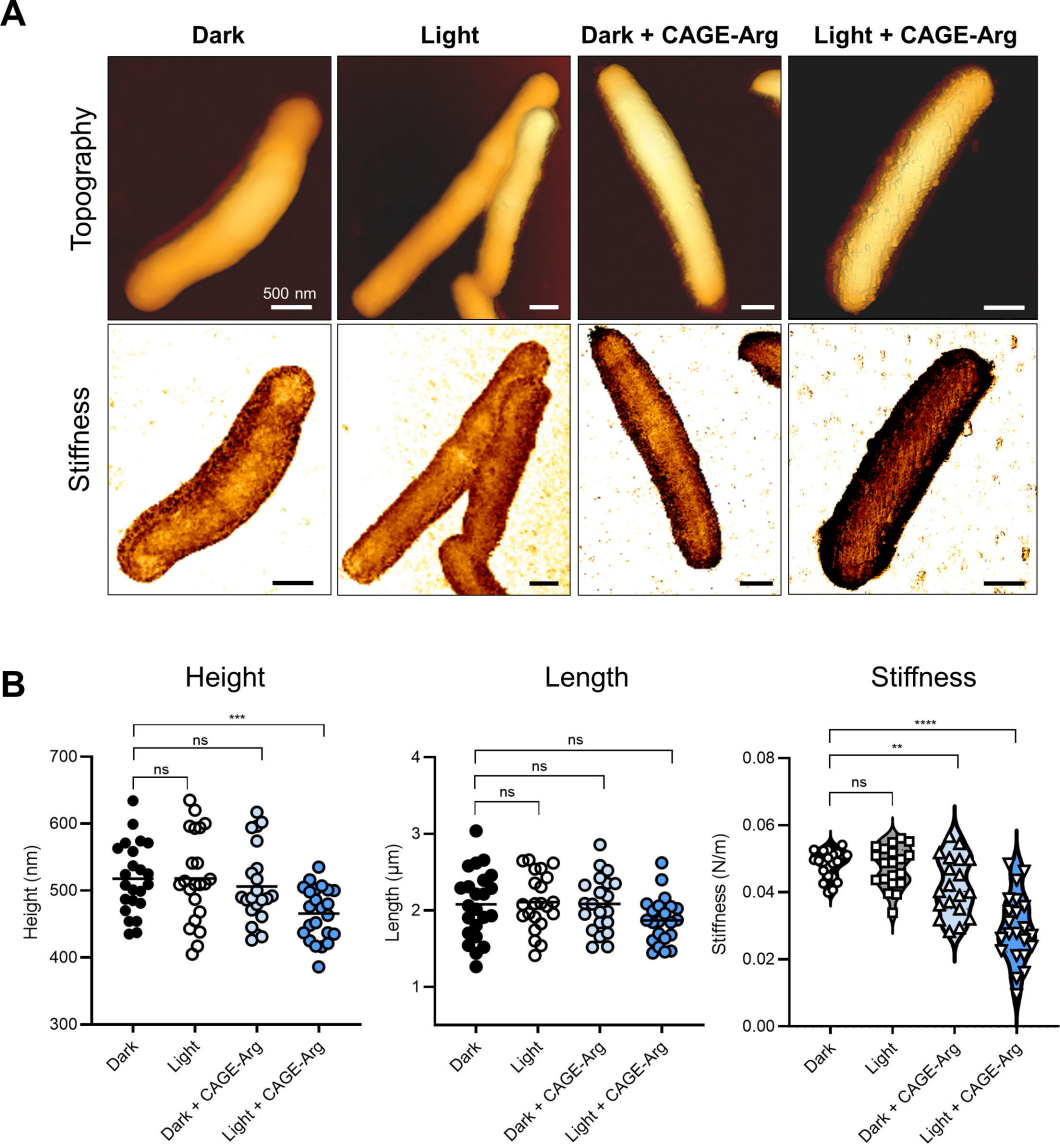
Quantitative imaging AFM analysis of *M. abscessus* treated in the presence of photoactivable CAGE-Arg. (A) Representative topographic (upper panel) and stiffness (lower panel) images of *M. abscessus* when submitted to four processing conditions: dark condition, light condition, CAGE-Arg (10 µM) treatment in dark condition, and CAGE-Arg (10 µM) treatment in light condition, respectively. (B) Bacterial height, length, and stiffness variations. Height and length were measured from topographic images using the calculated height channel at 80% of the force-distance curve (Dark, n = 23; Light, n = 22; Dark + CAGE-Arg, n = 21; Light + CAGE-Arg, n = 22). Stiffness measurements were extracted from the slope of the force-distance curves (Dark, n = 22; Light, n = 20; Dark + CAGE-Arg, n = 19; Light + CAGE-Arg, n = 22). *P*-values were calculated using an unpaired *t*-test. **P≤0.01; *** *P*≤ 0.001; ****P ≤ 0.0001; ns, non-significant.

The effect of treatment with CAGE-Arg on the morphology and mechanical properties of *M. abscessus* S was evaluated by atomic force microscopy (AFM). Bacteria were exposed to 10 µM CAGE-Arg in 0.9% NaCl for 30 min, either kept in the dark or irradiated for 10 min. The bacterial suspensions were next deposited on poly-L-lysine coated glass slides and imaged with a NanoWizard 4XP Bio-AFM (Bruker Nano GmbH, Berlin, Germany) in PBS at 20°C, using sharp nitride lever probes, cantilever C (SNL-C) probes (Bruker Nano GmbH, mean cantilever spring constant kcant  =  0.08 N/m). AFM analysis was carried out using force-distance spectroscopy (QI mode, Bruker Nano GmbH), which is based on force curves informing on the interaction between the AFM tip and the sample. These curves give access to the sample’s topography and stiffness simultaneously, upon a constant applied force (700 pN). Figure 2A shows common topography of a typical *M. abscessus* S (dark condition), ~0.5 µm in diameter and length distribution centered around 2 µm. The effective stiffness extracted from the force-distance curves was found in the range of 5.10^−2^ N/m, which fits with the typical range (10^−2^ N/m to 10^−1^ N/m) reported for various bacteria immobilized on substrates in comparable conditions (19–21). Irradiated bacteria (Fig. 2A) displayed similar morphology and no significant changes neither in dimensions nor in cell stiffness. While exposure to CAGE-Arg in the dark negatively impacted cell stiffness (Fig. 2B), pronounced changes were observed when *M. abscessus* was exposed to CAGE-Arg and irradiated, resulting in a mixture of intact and deflated cells harboring membrane wrinkles, protrusions, and extrusions (Fig. 2A). The treated cells appeared softer, indicating alteration of the bacterial membrane. Such dramatic changes in cell surface shape and roughness and alterations in their mechanical properties have been previously reported for bacteria treated with various porphyrins, peptides, or lipopeptides attacking the cell wall (22–24). These results provide evidence that combining light radiation and CAGE-Arg alter the membrane integrity and the mechanical properties of *M. abscessus*.

In summary, we provide here evidence that these new porphyrin-peptide cage-type conjugates, particularly CAGE-Arg, have potential for a photo-therapeutic use to treat *M. abscessus* skin infections and await further evaluations.

## Data Availability

All data will be made available upon request to the corresponding author.

## SUPPLEMENTAL MATERIAL

Supplemental material spectrum.00006-24-s0001.docx Fig. S1 and S2; Tables S1-S4.

ASM does not own the copyrights to Supplemental Material that may be linked to, or accessed through, an article. The authors have granted ASM a non-exclusive, world-wide license to publish the Supplemental Material files. Please contact the corresponding author directly for reuse.
