# Supplementary material for: Evaluation and activity of new porphyrin-peptide cage-type conjugates for the photoinactivation of Mycobacterium abscessus
Source: Microbiol Spectr. 2024 Apr 15;12(5):e00006-24. doi: 10.1128/spectrum.00006-24 (PMC11064497; doi:10.1128/spectrum.00006-24)
Supplement: Supplemental material — Fig. S1 and S2; Tables S1-S4. [file spectrum.00006-24-s0001.docx]

**SUPPLEMENTAL MATERIAL**

**Evaluation and activity of new porphyrin-peptide cage-type conjugates for the photoinactivation of *Mycobacterium abscessus***

Matthéo Alcaraz^1^, Sébastien Lyonnais^3^, Chandramouli Ghosh^4^, John Aguilera-Correa^1^,

Sébastien Richeter^5^, Sébastien Ulrich^4^ and Laurent Kremer^1,2,#^

^1^Centre National de la Recherche Scientifique UMR 9004, Institut de Recherche en Infectiologie de Montpellier (IRIM), Université de Montpellier, 1919 route de Mende, 34293, Montpellier, France.

^2^INSERM, IRIM, 34293 Montpellier, France.

^3^CEMIPAI, UAR3725, CNRS, Université de Montpellier, 1919 route de Mende, 34293, Montpellier, France.

^4^Institut des Biomolécules Max Mousseron (IBMM), Université of Montpellier, CNRS, ENSCM, Montpellier, France.

^5^Institut Charles Gerhardt Montpellier (ICGM), Université de Montpellier, CNRS, ENSCM, Montpellier, France.

^#^To whom correspondence should be addressed:

Tel: (+33) 4 34 35 94 47; E-mail: [laurent.kremer@irim.cnrs.fr](mailto:laurent.kremer@irim.cnrs.fr)

**Running title:** Photodynamic inactivation of *M. abscessus*

**Keywords:** *Mycobacterium abscessus,* photodynamic inactivation, photosensitizer, therapeutic activity, atomic force microscopy.

**Supplemental Materials and Methods**

**Effect of photoactivable compounds on *E. coli* biofilms**

To study photoactivable compounds on *E. coli* biofilms, 200 µL of tryptic soy broth (Merck, USA) supplemented with 1% glucose (w/v) (Merck, USA) (1) containing 4 × 10^6^ CFU/mL *E. coli* Stellar^TM^ (Takara bio) placed in each well of a flat-bottom untreated 96-well plate (MicroWell, ThemoFisher, USA) and incubated statically at 37 °C for 24 hrs. Afterward the incubation, the supernatant from each well was then removed, and 200 µL of sterile saline (Physiodose, Laboratoires Gilbert, France) with or without photoactivable compounds at 0.1 µM or 1 µM were deposited per well. The plate was incubated in the dark and at room temperature for 30 min and then irradiated with the 425 nm blue lamp for 5 or 10 min. After irradiation, biofilms were disaggregated by scrapping and homogenising with a 200 µL tip. Bacteria from the biofilms were diluted in a 10-fold bank dilution of PBS supplemented with 0.025% tyloxapol (v/v) and plated onto MacConkey agar plates without any sugar (BD, USA) to quantify CFU after overnight incubation at 37 °C. The experiment was performed by using three biological replicates and two technical replicates per condition (n = 6 per condition).

***M. abscessus* biofilms growing in a granuloma-like medium**

THP-1 monocytes were harvested, washed twice in PBS, lysed by freezing/thawing and pellets were resuspended in RPMI1640 with 2% heat-inactivated bovine serum at a concentration of 7.5×10^6^ cells/mL (2). *M. abscessus* was grown in 7H9^OADC/Ty^, seeded at an OD_600_=0.1, washed twice in PBS and resuspended in the leukocyte-based medium, dispensed (100 µL/well) in 96-well plates and incubated at 37 °C with 5% CO_2_, 0.9% O_2_ for 24 hrs to induce biofilms. The next day, supernatants were replaced by 200 µL of each PS (in 0.9% NaCl). Plates were incubated for 30 min at 37 °C in the darkness following irradiation with the blue light for 5 or 10 min. Serial dilutions in PBS with 0.025% tyloxapol were vortexed and sonicated for 3 min, plated onto LB agar and incubated at 37°C for 3 days prior to CFU counting.

**References**

1. Stepanović S, Vuković D, Hola V, Bonaventura GD, Djukić S, Ćirković I, Ruzicka F. 2007. Quantification of biofilm in microtiter plates: overview of testing conditions and practical recommendations for assessment of biofilm production by staphylococci. APMIS 115:891–899.

2. Ackart DF, Hascall-Dove L, Caceres SM, Kirk NM, Podell BK, Melander C, Orme IM, Leid JG, Nick JA, Basaraba RJ. 2014. Expression of antimicrobial drug tolerance by attached communities of *Mycobacterium tuberculosis*. Pathog Dis 70:359–369.

**Supplemental Figures**

***
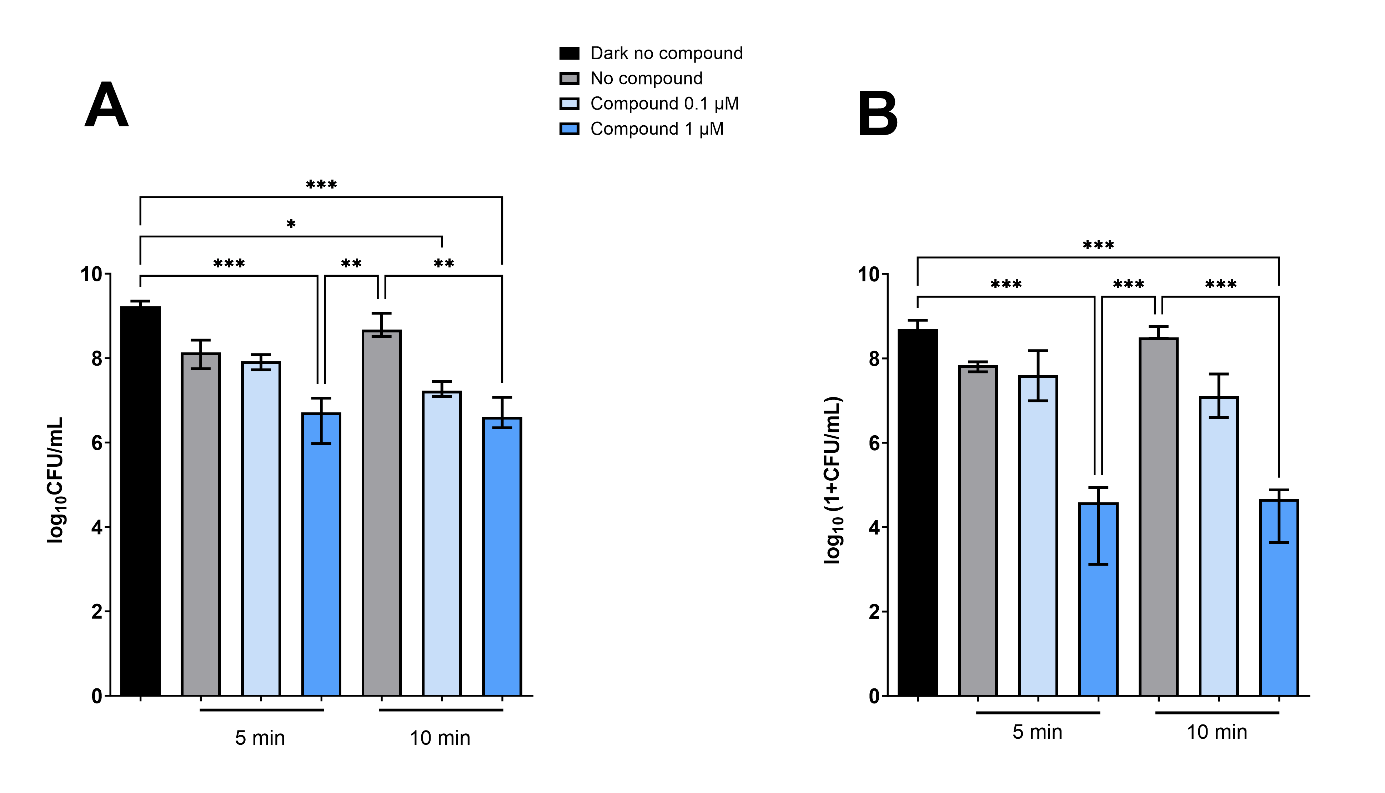
***

**Figure S1. Activity of photoactivable compounds on *E. coli* biofilms.** *E. coli* biofilms were exposed at two different concentrations of CAGE-Arg (**A**) or H_2_TMPyP (**B**) at 0.1 µM or 1 µM for 30 min prior to irradiation with a 425 nm blue lamp for 5 or 10 min. Quantification with CFU was determined immediately after irradiation. Untreated and/or unexposed biofilms were included as controls. Data of 3 independent experiments in duplicate were analyzed with Dunn test. The error bars represent the interquartile range. **P* ≤ 0.05; ***P* ≤ 0.01; ****P* ≤ 0.001.

**
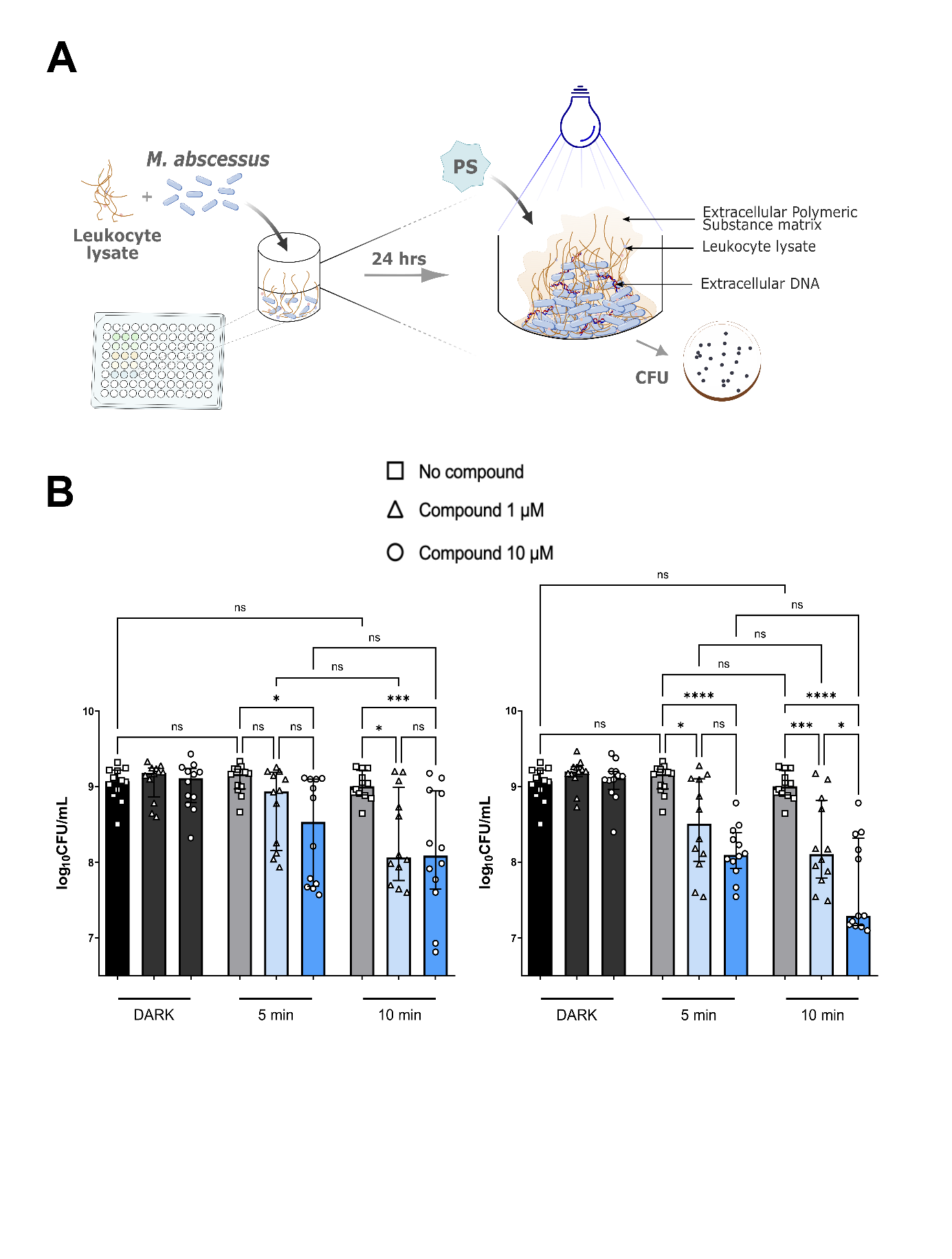
**

**Figure S2. Activity of CAGE-Arg and H2TMPyP on *M. abscessus* biofilms grown in granuloma-like medium. (A)** Scheme of leukocyte lysate-induced biofilms of *M. abscessus* (smooth variant) treated with PS compounds and irradiated with a blue light source. **(B)** *M. abscessus* S biofilms were exposed at 1 and 10 µM of either H2TMPyP (left) or CAGE-Arg (right) for 30 min prior to irradiation with a blue light source for 5 or 10 min, as indicated. CFU quantification was determined immediately after light irradiation. Untreated (black columns) and irradiated bacteria without PS (grey columns) were included as controls. The error bars represent the interquartile range. Data of 4 independent experiments in triplicates were analyzed using Mann-Whitney test **P* ≤ 0.05; ***P* ≤ 0.01; ****P* ≤ 0.001; *****P* ≤ 0.0001; ns: non-significant.

**Supplemental Tables**

**Table S1.** Quantity of *E. coli* (log_10_ CFU/mL) after irradiation with different concentration of compounds for different times. Data are expressed as mean ± standard deviation.

| **Irradiation time** | **0** | **5 min** | | | **10 min** | | |
| --- | --- | --- | --- | --- | --- | --- | --- |
| **Concentration (µM)** | **0** | **0** | **0.1** | **1** | **0** | **0.1** | **1** |
| H2TMPyP | 8.688±0.3877 | 8.669±0.4594 | 6.409±1.436 | 2.124±0.5056 | 8.648±0.4672 | 5.7±1.136 | 2.199±0.2814 |
| CAGE-Arg | 8.194±0.2853 | 8.146±0.2717 | 3.965±0.4406 | 2.511±0.7205 | 8.013± | 3.676± | 1.958±0.371 |
| CAGE-H | 8.526±0.2186 | 8.48±0.2109 | 7.418±0.6751 | 5.538±0.9143 | 8.527±0.2209 | 7.335±0.5453 | 5.38±0.9367 |

**Table S2.** Quantity of *E. coli* from biofilms (log_10_ [1+CFU/mL]) after irradiation with different concentrations of compounds for different times. Data are expressed as median (interquartile range).

| **Irradiation time** | **0** | **5 min** | | | **10 min** | | |
| --- | --- | --- | --- | --- | --- | --- | --- |
| **Concentration (µM)** | **0** | **0** | **0.1** | **1** | **0** | **0.1** | **1** |
| CAGE-Arg | 9.224(9.103-9.351) | 8.137(7.747-8.426) | 7.922(7.721-8.083) | 6.714(5.976-7.052) | 8.674(8.512-9.063) | 7.227(7.093-7.444) | 6.605(6.349-7.073) |
| H2TMPyP | 8.693(8.469-8.906) | 7.837(7.684-7.917) | 7.61(6.997-8.183) | 4.587(3.115-4.944) | 8.495(8.477-8.755) | 7.103(6.599-7.63) | 4.67(3.631-4.884) |

**Table S3.** Quantity of *M. abscessus* (log_10_ CFU/mL) after irradiation with different concentration of compounds for different times. Data are expressed as mean ± standard deviation.

| **Morphotype** | **Irradiation time** | **0** | **5 min** | | | **10 min** | | |
| --- | --- | --- | --- | --- | --- | --- | --- | --- |
|  | **Concentration (µM)** | **0** | **0** | **1** | **10** | **0** | **1** | **10** |
| Smooth | CAGE-H | 9.047±0.484 | 8.878±0.272 | 8.688±0.6933 | 8.224±0.9117 | 8.86±0.3043 | 6.216±0.5819 | 5.787±0.297 |
|  | CAGE-Arg | 8.489±0.7352 | 8.511±0.4142 | 7.132±0.4139 | 6.414±0.7782 | 8.013±0.4807 | 5.778±0.3331 | 4.225±0.8372 |
| Rough | CAGE-H | 8.631±0.291 | 8.545±0.3453 | 7.376±0.4284 | 7.002±0.675 | 8.552±0.3566 | 5.509±0.7434 | 5.482±0.8782 |
|  | CAGE-Arg | 7.917±0.4605 | 7.832±0.2556 | 5.89±0.7185 | 5.608±0.5208 | 7.672±0.4083 | 4.431±1.023 | 4.642±0.9546 |

**Table S4.** Quantity of smooth *M. abscessus* from biofilms grown in granuloma-like medium (log_10_ CFU/mL) after irradiation with different concentration of compounds for different times. Data are expressed as mean ± standard deviation.

| **Irradiation time** | **0** | | | **5 min** | | | **10 min** | | |
| --- | --- | --- | --- | --- | --- | --- | --- | --- | --- |
| **Concentration (µM)** | **0** | **1** | **10** | **0** | **1** | **10** | **0** | **1** | **10** |
| CAGE-Arg | 9.036 ± 0.2262 | 9.165 ± 0.1986 | 9.079 ± 0.2683 | 9.092 ± 0.1934 | 8.496 ± 0.6198 | 8.131 ± 0.3471 | 9.012 ± 0.1885 | 8.238 ± 0.5827 | 7.678 ± 0.6214 |
| H2TMPyP | 9.036 ± 0.2262 | 9.078 ± 0.2517 | 9.01 ± 0.3133 | 9.092 ± 0.1934 | 8.743 ± 0.5066 | 8.404 ± 0.6861 | 9.012 ± 0.1885 | 8.319 ± 0.6121 | 8.134 ± 0.7987 |
